# Extra-biliary complications during laparoscopic cholecystectomy: How serious is the problem?

**DOI:** 10.4103/0972-9941.40990

**Published:** 2008

**Authors:** Arshad M Malik, Abdul Aziz Laghari, Qasim Mallah, Fazila Hashmi, Ubaid Sheikh, K Altaf Hussain Talpur

**Affiliations:** Department of Surgery, Liaquat University of Medical and Health Sciences, Jamshoro, Hyderabad, Pakistan

**Keywords:** Extra-biliary complications, lap chole, morbidity, mortality

## Abstract

**Objective:**

To deteremine the incidence, nature and management of extra-biliary complications of laparoscopic cholecystectomy.

**Materials and Methods:**

This study presents a retrospective analysis of extra-biliary complications occuring during 1046 laparoscopic cholecystectomies performed from August 2003 to December 2006. The study population included all the patients with symptomatic gallstone disease in whom laparoscopic cholecystectomy was performed. The extra-biliary complications were divided into two distinct categories: (i) Procedure related and (ii) Access related.

**Results:**

The incidence of access-related complications was 3.77% and that of procedure-related complications was 6.02%. Port-site bleeding was troublesome at times and demanded a re-do laparoscopy or conversion. Small bowel laceration occurred in two patients where access was achieved by closed technique. Five cases of duodenal and two of colonic perforations were the major complications encountered during dissection in the area of Calot's triangle. In 21 (2%) patients the procedure was converted to open surgery due to different complications. Biliary complications occurred in 2.6% patients in the current series.

**Conclusion:**

Major extra-biliary complications are as frequent as the biliary complications and can be life-threatening. An early diagnosis is critical to their management.

## INTRODUCTION

Laparoscopic cholecystectomy is considered superior to open cholecystectomy in terms of morbidity, cosmesis and rate of complications.[1–3] There are, however, other studies which report an increased rate of complications during laparoscopic cholecystectomy compared to open cholecystectomy.[4–8] Biliary complications are reported in many studies.[9–15] The extra-biliary complications do occur with almost the same frequency and severity but tend to be under-reported in the literature.[16] The extra-biliary complications can be access-related or procedure-related. Different techniques of abdominal access are described but none has been found to be superior in terms of preventing access-related injuries.[17] Although these complications are not as common as they were in the past, but are still a major source of morbidity associated with laparoscopic cholecystectomy. Fuller *et al.*[18] reported laparoscopic cholecystectomy as a procedure most frequently associated with both fatal and non-fatal trocar-related injuries. We report extra-biliary complications in this study with emphasis on their incidence, severity and management.

## MATERIALS AND METHODS

This is a reterospective analytical study of 1046 patients in whom laparoscopic cholecystectomy was performed in the Department of Surgery, Liaquat University Hospital, Jamshoro and private hospitals, during August 2003 to December 2006. The cases were operate upon by five surgeons with different levels of experience. The study population included all the patients with symptomatic gallstone disease regardless of their age and gender. All the patients were operated by the classical four-port technique while few amendments were made according to the situation, such as placing an additional port etc. In 275 cases, pneumoperitoneum was created using a Verress needle and in 771, by a technique of direct trocar insertion.

The choice of method for creation of pneumoperitoneum was solely decided by the operating surgeon. Details of each patient were recorded on a proforma. The complications were divided into access-related complications and procedure-related complications. The results were analyzed statically using SPSS version 10.

## RESULTS

There were 86 (8.22%) males and 960 (91.77%) females with a male to female ratio of 1:11. The age ranged from 20 to 74 years with a mean age of 45.37 years as shown in Table 1. Incidence and nature of various complications encountered are shown in Table 2A and B. Simple gallstone disease was found in 815 (77.91%) patients while remaining 231 (23%) patients had complicated gallstone disease. Direct trauma to superior epigastric vessels during trocar insertion caused torrential bleeding in seven patients of which five were converted to open surgery. In the remaining patients, the bleeding occurred either from injury to inferior epigastric vessels or muscular abdominal vessels, which was effectively arrested by suture ligation, external or laparoscopic diathermy and temponade. The procedure-related complications mostly occurred in patients with complicated gallstone disease like empyema or acute cholecystitis. A low threshold for conversion during the early phase of learning may save precious lives as most coversions are done for life-threatening complications as shown in Table 3. All of the duodenal and colonic perforations were identified during the operation while small bowel perforation was identified on third post-operative day with the development of peritonitis. The average hospital stay was 48 h and there was no mortality in this series.

**Table 1:** Age of patient, gender of patient and cross-tabulation

		Gender of patient		Total	
				
		Males	Females	
Age of patient	20-30	5	60	65
	31-40	40	173	213
	41-50	20	567	587
	51-60	16	116	132
	61-70	3	36	39
	71-80	2	8	10
Total		86	960	1046

**Table 2A:** Access-related complicatons (n = 38)

Complication	No. of patients	Percentage
port site bleedig	13	1.24
Small bowel laceration	2	0.19
Sub-cutaneous emphysema	17	1.62
Retained stones in port causing port sepsis	3	0.28
Small bowel puncture	1	0.09
Ascending colon leceration	2	0.19

**Table 2B:** Procedure-related complications (n = 63)

Complication	No. of patients	Percentage
Sub-capsular liver hematoma	9	0.86
Duodenal perforation	5	0.47
Bleeding through Gallbladder bed	11	1.05
Colon perforation	2	0.19
Spillage of stones in the peritoneal cavity	17	1.62
Bleeding through cystic artery	19	1.81

**Table 3:** Conversion to open cholecystectomy and the underlying causes

Complication	No. of Patients	Conversion to open cholecystectomy	Percentage
Duodenal perforation	5	5	0.5
Port site bleeding	13	5	0.5
Bleeding from cystic artery	19	08	0.8
Colon perforation	2	2	0.2
Small bowel perforation	1	01	0.1
Total	40	21	2.0

## DISCUSSION

The extra-biliary complications reported in this series are either access-related or procedure-related. Access-related complications are common despite various changes made in the access techniques. Hashizume and Sugimachi[19] have reported trocar injuries to bowel and major blood vessels to be as high as 1% and most of them have occurred during the insertion of the first tocar. Schafer *et al.*[20] in their study report a similar result. Blind trocar insertion and access by verress needle remain the important causes of complications as reported by many authors. In our experience, most common access-related complications were port-site bleeding and extra-peritoneal insufflations resulting in surgical emphysema of varying degrees. Direct trauma to superior epigastric vessels can lead to uncontrollable bleeding and ultimate conversion. Gaining access by closed technique has a complication rate in the range of 0.2–0.3% as reported by Loffler and Pent.[21] On the other hand, open technique of trocar insertion has promising results and seems to have reduced the access-related major vessel injury and mortality rate.[22,23] Trocar insertion under vision through avascular planes and a thorough inspection of the ports before deflation of the abdomen can reduce port site bleeding. Mayo *et al.*[24] have made a similar recommendation in their study. Undue thrusting force during first trocar insertion is likely to cause bowel injury. Adequate manual lifting of the abdominal wall during insertion is very helpful and gives good safety. Illuminating the abdominal wall by telescope may display the vessels and secondary ports may be created safely. Subcutaneous emphysema usually occurs due to leakage of gas from the site of trocar insertion and is likely when patient is obese and gas is insufflated through a misdirected Veress needle. This may require manual pressure on abdominal wall to evacuate the gas. This is consistent with other similar studies.[25] Previous operations may make abdominal access difficult and liable to produce bowel injury. Access-related bowel injuries were found more common with closed technique of abdominal access. This is similar to results of other similar studies.[26–28] We report 6% overall procedure-related problems in this series of which 15 (1.43%) were serious enough to demand conversion to open procedure. Procedure-related complications are more likely to occur when there is history of repeated attacks of acute cholecystitis leading to distortion of anatomy of Calot's triangle. Colonic perforation was another serious procedure-related complication and occurred in two patients, both of which were converted. These procedural injuries to gastrointestinal tract are associated with a high mortality rate as indicated by various studies.[26–29] The duodenal injuries in our study were caused during difficult dissection in the Calot's triangle. This is consistent with other reports[30] and usually results when dissection is continued in a totally obscured field. Patience, displaying of anatomy and identification of structures before cutting or applying clips are vital to safe outcome.

## CONCLUSION

Extra-biliary complications during laparoscopic cholecystectomy are almost equally common and can prove to be lethal if not identified and managed during the operation. Patience and low threshold for conversion in difficult cases can substantially decrease morbidity and mortality.
